# Structural Insights into the Evolution of a Sexy Protein: Novel Topology and Restricted Backbone Flexibility in a Hypervariable Pheromone from the Red-Legged Salamander, *Plethodon shermani*

**DOI:** 10.1371/journal.pone.0096975

**Published:** 2014-05-21

**Authors:** Damien B. Wilburn, Kathleen E. Bowen, Kari A. Doty, Sengodagounder Arumugam, Andrew N. Lane, Pamela W. Feldhoff, Richard C. Feldhoff

**Affiliations:** 1 Department of Biochemistry and Molecular Biology, University of Louisville, Louisville, Kentucky, United States of America; 2 J.G. Brown Cancer Center, University of Louisville, Louisville, Kentucky, United States of America; The University of Tokyo, Japan

## Abstract

In response to pervasive sexual selection, protein sex pheromones often display rapid mutation and accelerated evolution of corresponding gene sequences. For proteins, the general dogma is that structure is maintained even as sequence or function may rapidly change. This phenomenon is well exemplified by the three-finger protein (TFP) superfamily: a diverse class of vertebrate proteins co-opted for many biological functions – such as components of snake venoms, regulators of the complement system, and coordinators of amphibian limb regeneration. All of the >200 structurally characterized TFPs adopt the namesake “three-finger” topology. In male red-legged salamanders, the TFP pheromone Plethodontid Modulating Factor (PMF) is a hypervariable protein such that, through extensive gene duplication and pervasive sexual selection, individual male salamanders express more than 30 unique isoforms. However, it remained unclear how this accelerated evolution affected the protein structure of PMF. Using LC/MS-MS and multidimensional NMR, we report the 3D structure of the most abundant PMF isoform, PMF-G. The high resolution structural ensemble revealed a highly modified TFP structure, including a unique disulfide bonding pattern and loss of secondary structure, that define a novel protein topology with greater backbone flexibility in the third peptide finger. Sequence comparison, models of molecular evolution, and homology modeling together support that this flexible third finger is the most rapidly evolving segment of PMF. Combined with PMF sequence hypervariability, this structural flexibility may enhance the plasticity of PMF as a chemical signal by permitting potentially thousands of structural conformers. We propose that the flexible third finger plays a critical role in PMF:receptor interactions. As female receptors co-evolve, this flexibility may allow PMF to still bind its receptor(s) without the immediate need for complementary mutations. Consequently, this unique adaptation may establish new paradigms for how receptor:ligand pairs co-evolve, in particular with respect to sexual conflict.

## Introduction

Pheromone signaling is an essential means of communication for many animals to acquire information for a vast array of qualities on other individuals, including species, gender, reproductive status, and disease state [1]–[4]. For nearly all characterized systems, pheromone molecules are perceived via specialized receptors within a target’s olfactory system, and in turn elicit specific, pre-programmed behavioral and/or endocrine responses [5]. For more than 50 years, the earliest and best characterized pheromone systems have been those in insects, commonly employing small, volatile odorants as chemoattractants [1], [6]. The evolution of such systems has been extremely difficult to study, as these odorant molecules are generally the products of complex enzymatic cascades [7]. In contrast, multiple vertebrate systems utilize peptide or protein pheromones to act as chemical signals; as direct gene products, these pheromones are more tractable to both biochemically synthesize and investigate their evolutionary origins [8]. Because pheromones are ligand molecules that bind to target receptors, it is critical to deduce the 3-dimensional structure of pheromone molecules in order to address how different selective mechanisms may be acting in a co-evolutionary receptor-ligand framework. To date, protein structures have only been determined for two mouse pheromones: a major urinary protein (MUP) that affects male aggressive behavior [9], [10] and the male sex pheromone exocrine gland-secreting peptide 1 (ESP1) [11], [12]. Consequently, little is known about the structural evolution of pheromones in non-mammalian vertebrates.

Over the past 25 years, plethodontid salamanders have served as a valuable model for investigating the mechanisms by which protein pheromones regulate behavior and reproductive success [8], [13]. As basal tetrapods, salamanders are an excellent model to study the origins of terrestrial pheromone signaling in vertebrates. For more than 100 million years, plethodontid salamanders have utilized a unique courtship behavior, termed tail straddling walk, to coordinate insemination and facilitate mating success [14]. In the species *Plethodon shermani*, during tail straddling walk, male salamanders will periodically deliver non-volatile, proteinaceous courtship pheromones to the female by “slapping” an enlarged gland on his chin (the mental gland) to the female’s nares [15], [16]. After a male has applied pheromone to the female that he is courting, the protein molecules diffuse into the female’s olfactory chamber where they bind to receptors on vomeronasal neurons, which project to specific regions of the brain, and influence the female’s mating behavior [17]–[19]. It is noteworthy that these pheromones are applied after courtship has initiated, and function to regulate female mating behavior: they are not chemoattractants [16]. Chemical analysis of the *P. shermani* pheromone composition revealed two major components: Plethodontid Receptivity Factor (PRF), a 22-kDa protein related to IL-6 cytokines, and Plethodontid Modulating Factor (PMF), a 7-kDa protein related to the three-finger protein (TFP) superfamily [15], [20], [21]. Both PRF and PMF persist as multi-isoform blends; however, compared to PRF with only 3 isoforms which share ∼95% identity, individual male salamanders synthesize more than 30 unique PMF isoforms with ∼30% amino acid identity [20]. Multiple studies of molecular evolution have demonstrated that PMF is under pervasive positive selection, presumably in response to sexual selection from co-evolving female receptors [20], [22].

The TFP superfamily, of which PMF is a member, includes many diverse proteins such as snake neuro- and cytotoxins [23], [24], regulators of the complement system [25], membrane receptors in mammalian tissue re-organization [26], and factors that facilitate amphibian limb regeneration [27]. One central idea in the field of protein structural biology is that, throughout protein evolution, structure is generally more highly conserved than sequence, often as a consequence of functional requirements that promote purifying selection [28]. In support of this, while more than 90,000 structures have been deposited in the PDB, all of these proteins adopt a relatively small number of topological folds (∼1300 in CATH) [29], [30]. Furthermore, studies in evolutionary biochemistry further suggest that only a few high-impact mutations on these conserved topologies is necessary for the evolution of novel functions [31]. The TFP superfamily well exemplifies this phenomenon. Establishing a well-resolved TFP phylogeny has been difficult, as homologs from different species share little amino acid similarity and are difficult to align [27], [32]; however, the defining feature of this superfamily is the conserved protein structure of two parallel β-sheets (2- and 3-stranded) arranged in a “three-finger” shape. Importantly, this shape is highly stabilized by 8 conserved cysteine residues that adopt a canonical disulfide bonding pattern (**1–3**, **2–4**, **5–6**, **7–8**). To date, more than 200 TFP structures have been solved by X-ray crystallography or multidimensional NMR, and all share this canonical disulfide bonding pattern and three-finger shape [27].

PMF has many unique characteristics compared to nearly all other TFPs, despite preservation of the 8 conserved cysteine residues and their relative spacing. First, in plethodontid salamanders, PMF has been subjected to exacerbated gene duplication and pervasive positive selection, compared to most organisms where TFPs with particular functions are found as single gene copies, and snake venom glands have been specially noted for having up to 5 isoforms of different toxins [23], [33]. Second, while most TFPs carry a net positive charge, nearly all *P. shermani* PMFs are highly negatively charged (mean charge = −9.1). Third, as the only pheromone TFP, rather than being under natural selection like other TFPs, PMF was novel as the first identified TFP under sexual selection. Despite extensive sequencing and proteomic analyses [20], it remained unclear how the evolution of PMF hypervariability in response to sexual selection might influence the archetypal TFP structure. Therefore, in order to better characterize the structure:function relationships of the PMF pheromone family, the aim of this study was to determine the complete 3D structure of the most abundant PMF isoform (Isoform G; Genbank Accession #JF274292).

## Materials and Methods

### Ethics Statement

Methods and animal care were approved by Oregon State University’s Institutional Animal Care and Use Committee (ACUP 3007 to L.D. Houck). Animals were anesthetitized prior to surgery using diethyl ether, minimizing any pain, and post-surgical survival rate was >99%. All salamanders were collected under permits obtained from the North Carolina Wildlife Resources Commission.

### Reagents

All oligonucleotides were synthesized by Integrated DNA Technologies (Coralville, IA). Accuprime High Fidelity (HF) *Taq* Polymerase System, the EasySelect Pichia Expression Kit (including the vector pPICZαA), Zeocin, ultra-pure agarose, and TOP10 chemically competent *E. coli* were purchased from Invitrogen (Carlsbad, CA). All restriction enzymes, T4 DNA Ligase, and additional PCR supplies were purchased from New England Biolabs (Ipswich, MA). GFX gel band purification system was purchased from GE Healthcare (Piscataway, NJ). QIAquick PCR purification system was purchased from Qiagen (Valencia, CA). Sep-Pak Light C-18 cartridges were purchased from Waters Division (Milford, MA). Centriprep ultrafiltration units were purchased from Millipore (Billerica, MA). Trypsin, trifluoracetic acid (TFA), and all salts were purchased from Sigma-Aldrich (St. Louis, MO). Yeast media reagents, Whatman DEAE cellulose, and acetonitrile (ACN) were purchased from Fisher Scientific (Pittsburgh, PA).

### High Performance Liquid Chromatography (HPLC)

High resolution strong-anion exchange HPLC (Mono Q; Pharmacia, Piscataway, NJ), reverse phase-HPLC (RP-HPLC) (C-18; Grace Davison Discovery Sciences, Deerfield, IL), and size exclusion chromatography (G-75 Superfine; Pharmacia, Piscataway, NJ) was accomplished on a 2695 Alliance HPLC System equipped with a 2487 dual wavelength absorbance detector and Empower software (Waters Division, Milford, MA). The strong anion exchange column (0.5×5.5 cm) was eluted at 1 mL/min with a NaCl gradient in 50 mM Tris/HCl buffer, pH 8.0. The C-18 column (0.46×15 cm) was eluted with an ACN gradient in 0.1% (v/v) TFA at 1 mL/min. The G-75 column (1.6×15.5 cm) was isocratically eluted at ∼10 mL/hr with 0.5×phosphate-buffered saline.

### Mass Spectral Analysis

Picomole quantities of PMF-G were provided to the University of Louisville Biomolecular Mass Spectrometry Core Laboratory. Intact protein mass was determined by electrospray ionisation mass spectrometry (ESI-MS) using a Q-TOF API-US (Waters Division, Milford, MA), while proteolytic fragment fingerprints were acquired by liquid chromatography tandem mass spectroscopy (LC/MS-MS) using a LTQ Orbitrap XL (Thermo Scientific, Waltham, MA). SEQUEST software (Thermo Scientific, Waltham, MA), MassMatrix v.1.3.2 [34], or custom Python scripts built around Extract-MSn (Thermo Scientific, Waltham, MA) were used for all peptide analyses. The average masses of intact proteins and monoisotopic masses of peptides were matched to theoretical average or monisotopic masses, respectively. Predicted intact masses were adjusted by 1.0078 Da per cysteine to account for the protons displaced in disulfide bonds.

### Purification of Natural PMF-G

*P. shermani* males were collected during their breeding season from a single site in Macon Co., North Carolina, USA (35°10′48″ N, 83°33′38″ W). Males were anesthetized in a mixture of 7% (v/v) diethyl ether in water. Pheromones were extracted following the methods of Houck et al.^21^. Approximately 100 glands were excised and pheromones extracted with 0.8 mM acetylcholine chloride in Amphibian Ringer’s Solution for ∼60 minutes, centrifuged at 14,000×*g* for 10 minutes, the supernatant collected, and the centrifugation repeated before storage at −80°C. PMF isoform G was purified from the whole pheromone extract using the methods described in Wilburn et al. [20].

### Preparation of rPMF-G Expression Strain

The *P. pastoris* codon optimized sequence for the most abundant PMF isoform, PMF-G (Genbank Accession # JF274292), was predicted by web-based software from IDT (Coralville, IA). Six overlapping and complementary oligonucleotides based on the sequence were prepared, and used in assembly PCR based on the methods of Stemmer et al. [35]. Purified PCR products were ligated to the vector pPICZαA and cloned into TOP10 chemically competent *E. coli* using standard procedures. Plasmid DNA from Zeocin-resistant clones was purified, sequenced to validate the construct, linearized by restriction digest with *Sac*I, and transformed into *P. pastoris* strains KM71H and GS115 using the EasySelect Pichia Expression Kit. Zeocin-resistant *P. pastoris* clones were screened for recombination by colony PCR using primers flanking the AOXI locus. Two positive clones from each *P. pastoris* strain were used for small-scale protein expression following the manufacturer’s protocols with products analyzed by SDS-PAGE.

### Large Scale Preparation of rPMF-G

For each preparation, 400 mL BMGY (100 mM potassium phosphate, pH 6.0, 2% peptone, 1% yeast extract, 1.34% YNB, 4×10^−5^% biotin, 1% glycerol) was inoculated with strain KM71H clone 1 and incubated at 29°C with shaking at 275 rpm for ∼40 hours. Cells were collected by centrifugation, resuspended in 1 L BMM (100 mM potassium phosphate, pH 6.0, 1.34% YNB, 4×10^−5^% biotin, 1% methanol) with an initial optical density (600 nm) of 0.5, and allowed to continue shaking for ∼72 hours with 1% methanol added every 24 hours. Cultures were then centrifuged at 4000×*g* for 20 minutes, the supernatant collected, filtered, and pumped over a 25 mL DEAE-cellulose column pre-equilibrated in 50 mM NaCl/50 mM Tris/HCl, pH 8 at ∼0.8 mL/min. The column was then washed with 150 mL 100 mM NaCl/1% Triton X-100/1 M Urea/50 mM Tris-HCl, pH 8 and the sample eluted in 75 mL 300 mM NaCl/1% Triton X-100/1 M Urea/50 mM Tris-HCl. The sample was twice diluted (1∶4) with 1% Triton X-100/1 M Urea/50 mM Tris-HCl, pH 8 and concentrated to ∼2.5 mL using a YM-3 Centriprep. The sample was then treated with trypsin in a 1∶40 mass ratio overnight at 37°C, products separated by strong anion exchange HPLC (linear gradient: 0–500 mM NaCl in 50 mM Tris-HCl, pH 8 at 10 mM NaCl/min), fractions collected, pooled, and further purified by two rounds of RP-HPLC (linear gradient: 0–40% ACN at 1% ACN/min).

### Validation of rPMF-G Structure

Monoisotopic intact protein masses were obtained for both PMF-G and rPMF-G using ESI-MS. Both proteins were reduced with dithiothreitol (DTT) and alkylated with iodoacetamide (IAA) prior to mass acquisition by ESI-MS. Tryptic fragments were sequenced by LC/MS-MS. Far-UV circular dichroism (CD) spectra (185–260 nm) were acquired for native and rPMF-G by averaging 5 scans across a 0.1-cm path at 0.2 nm intervals using a Jasco J-810 Spectropolarimeter, and curves processed using the R function loess.smooth with smoothness parameter 0.05; α-helix and β-sheet content were estimated using the web application K2D3 [36]. The state of oligomerization was determined by analytical ultracentrifugation using a Beckman XLA analytical ultracentrifuge. The C(S) profile showed a monodisperse sample with an S value consistent with a monomer. The molecular weight derived from the sedimentation coefficient, diffusion coefficient and calculated partial specific volume was within 10% of the expected mass.

### PMF Partial Reduction Analysis

Partial cystine reduction was accomplished at low pH in order to prevent disulfide bond reformation and scrambling. Both natural and rPMF-G were subjected to restricted reduction using a trialkyphosphine (TCEP; tris-[-2-carboxyethyl]-phosphine; Pierce) at pH 3 in 0.1% TFA for 20 minutes at room temperature followed by immediate injection onto the C-18 RP-HPLC column at pH 2.2 (linear gradient 17.5% to 35% ACN at 0.35% ACN/min). Individual peaks corresponding to the cleavage of 0, 1, 2, 3 or 4 disulfide bonds were collected by hand. Volume and ACN concentration were reduced by incomplete lyophilization. Samples were rapidly alkylated by dropwise addition of the protein solution into 500 µL IAA (5 mM in 100 mM Tris, pH 8) while vortexing for ∼1 min before the pH was lowered by addition of 500 µL 5% formic acid. Alkylated samples were purified using a C18 Zip-tip, divided into 4 aliquots, and diluted with 100 mM ammonium bicarbonate. DTT (7.5 mM) was added to half of the samples, and all samples were subjected to overnight proteolysis with either chymotrypsin or AspN such that all reduction/protease combinations were performed. Peptide fragments were purified by C18 Zip-tip, and analysed by LC/MS-MS.

### NMR Structural Studies

Milligram quantities of ^15^N-labeled rPMF-G were prepared using the methods above with 1.5 g/L ^15^N-ammonium sulfate (99%) added to the BMM expression media, and prepared at ∼2.2 mM in 50 mM KCl/10 mM Na_2_HPO_4_, 90% H_2_O/10% D_2_O, pH 7. NMR spectra were recorded at 18.8 T on a Varian Inova spectrometer equipped with a 5 mm inverse triple resonance pfg probe at 20°C. Spectra were processed using NMRPipe [37]. NMR assignments were obtained using a combination of 2D/3D experiments (using ^1^H and ^15^N, with natural abundance levels of ^13^C): TOCSY-^15^N-HSQC, 2D-TOCSY, 2D-COSY, H^15^NCO, ^15^N-HSQC, ^13^C-HSQC, NOESY-^15^N-HSQC, and 2D-NOESY. All NMR spectra were acquired in phase sensitive mode with solvent suppression by Watergate [38]. Spectra were analyzed using SPARKY (T. D. Goddard and D. G. Kneller, SPARKY 3, University of California, San Francisco). Near complete assignment of all backbone atoms (98% non-proline ^1^H_N_, 91% ^15^N, 100% ^1^H_α_, 96% ^13^C_α_, and 46% ^13^C_O_) and 97% side-chain ^1^H atoms were obtained. Structure calculations were performed using CYANA [39], [40] with automatic assignment to integrated 2D-NOESY and NOESY-^15^N-HSQC peaks. Dihedral angle restraints were obtained using predictions from TALOS+ [41]. H_N_ exchange rate was measured by lyophilizing rPMF-G, the sample resolubilized in 99.99% D_2_O, and ^15^N-HSQC spectra recorded every hour for 24 hours. Amide groups with half lives greater than 2.5 hours were examined for possible H-bonding partners, and defined using the CYANA hbond function if the distance between the groups was less than 2.5 Å. C_α_ and C_β_ chemical shifts suggest all cysteine residues are oxidized and disulfide bonded [42]; alternative disulfide patterns (for both candidate PMF-G patterns as well as the canonical TFP pattern) were included during CYANA constraint calculation, and the **1–2, 3–6, 4–5, 7–8** pattern yielded the lowest average target score and fewest consistent constraint violations, strongly supporting that it is the correct disulfide bonding pattern. The final ensemble of 20 out of 100 structures did not contain structural or van der Waals violations >0.30 Å. For dihedral angles, there were no violations >3.2°, and 88.9% of all dihedral angles were found in the most favored regions of the Ramachandan plot, with 9.0% in the additionally allowed regions and the remaining 2.1% in the generously allowed regions. Spin-lattice (longitudinal) relaxation rate constants (R_1_), spin-spin (transverse) relaxation rate constants (R_2_), and ^15^N{^1^H} steady-state heteronuclear NOEs of the backbone ^15^N nuclei were measured at 18.8 T and 293 K. Delay values used were 10, 30, 50, 90, 130, 170, and 210 ms for R_2_ experiments, and 10, 80, 150, 300, 500, 750, and 1000 ms for R_1_ experiments, all with a recovery delay of 5 seconds. For ^15^N{^1^H} NOE measurements, two spectra were acquired with or without 5 seconds of proton saturation during the recovery delay, with both the saturated and unsaturated experiments having a relaxation delay of 5 seconds. All NMR data were deposited in the BMRB (19660), and the structural ensemble deposited in the PDB (2 mhy).

### Structural Analysis

All 3D protein models were produced in PyMOL (v1.3, Schrodinger, LLC), and regular secondary structure defined using the DSS function in PyMOL. Surface renderings with charge distribution are color coded according to amino acid type: acidic (red: Glu, Asp), basic (blue: Lys, Arg, His), hydrophilic (magenta: Ser, Thr, Gln, Asn, Gly), nonpolar (green: Ala, Leu, Ile, Val, Phe, Tyr, Trp, Met, Pro), or cysteine (yellow: Cys). PMF structural comparisons were made to a representative TFP (short chain neurotoxin from *Naja nigricollis*, 1IQ9); length and charge calculations for structurally characterized TFPs were based on results from ScanProsite at the ExPASy server with query “C-x(5,30)-C-x(2,10)-C-x(10,30)-C-x(2,20)-C-x(5,30)-C-C-x(4)-C-N” (method adapted from Garza-Garcia et al. [27]). H_N_ exchange half-lives were calculated by non-linear least-squares regression of the peak integration versus time for H-D exchange experiments. R_1_ and R_2_ rate constants were determined by similar non-linear least squares regression of the exponential decay curves, and standard deviations were calculated from the curvature matrix. Confidence intervals were determined for the relaxation rate constant of each residue. The NOEs were calculated as the ratio of the peak volumes in the saturated and non-saturated spectra. Reduced spectral density mapping was performed as described in McIntosh et al. [43]. The random coil index for PMF-G was calculated using chemical shift values submitted to the RCI server [44]. PMF sequence variability for all Class I PMFs (Genbank accession #JF274283–274351) was calculated using the protein variability server (PVS) [45], with likelihood of positive selection based on Bayes empirical Bayes results for M2A site specific PAML models from Wilburn et al. [20]. Homology modeling was conducted using Rosetta 3.4 [46]: sequences for additional PMF isoforms were aligned to PMF-G using ClustalW [47], alignable elements of the new isoform sequence superimposed on the peptide backbone of the lowest energy PMF conformer, the disulfide bonding pattern fixed to that of PMF-G, insertion loops built using the loopmodel function, and fastrelax applied to minimize the energy of the resulting model. Ten thousand models were generated per isoform, cluster analysis performed with the cluster radius automatically determined, and the lowest energy structure of the most abundant cluster reported.

## Results

### Disulfide Bond Characterization of PMF-G

**Figure 1:**
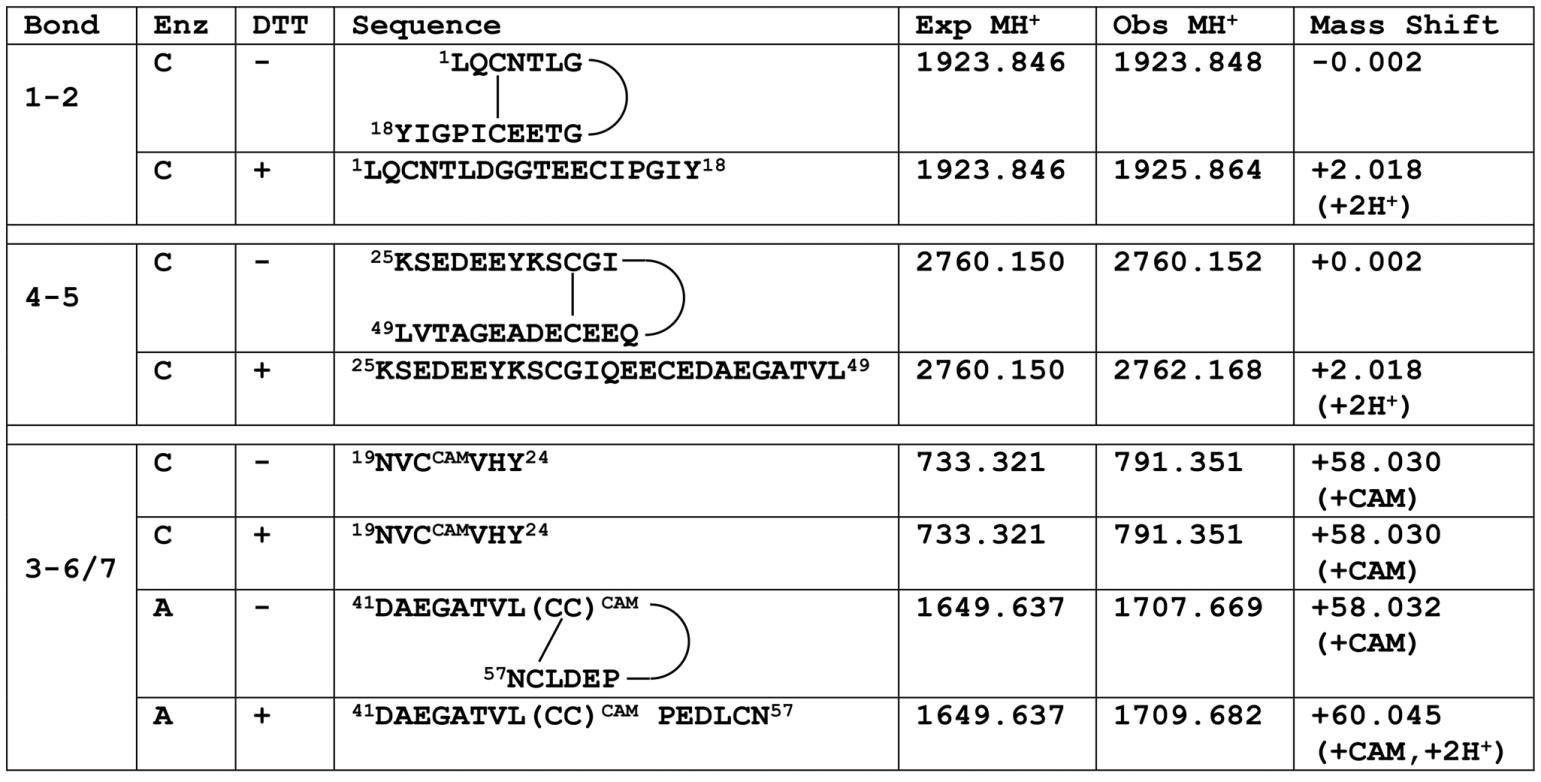
Summary of mass spectral analysis in PMF-G disulfide bonding pattern determination. Mass spectral analyses was performed on the three-disulfide species of PMF-G purified by RP-HPLC. Differential treatment included proteolytic enzyme (Enz; chymotrypsin [C] or AspN [A]), reduction with dithiothreitol (DTT), and alkylation with iodoacetamide (addition of a carboxyamidomethyl (CAM) group). Observed monoisotopic masses were compared to theoretical masses with no free sulfhydryls, and mass shifts used to determine peptide modification. All assignments were confirmed by analysis of the fragmented ion series.

As a member of the TFP superfamily, PMF was expected to adopt the canonical TFP disulfide bond pattern. Initial experiments relied on natural PMF-G purified from *P. shermani* whole pheromone extract through a series of chromatographic separations [20] (Figure S1). Analysis by mass spectrometry (MS) confirmed that all 8 cysteine residues were disulfide bonded (Figure S2). However, due to its small size and high disulfide density, PMF-G was extremely protease resistant, and initial efforts to characterize the disulfide bonds by MS with proteolytic digestion yielded ambiguous data that suggested a non-canonical pattern. Based on the methods of Gray [48], PMF-G was next subjected to partial disulfide bond reduction, and the resulting protein species, having different numbers of reduced disulfides, were separated by reverse phase high performance liquid chromatography (RP-HPLC) at pH 2.2 to prevent re-oxidation (Figure S3). Extensive experimentation confirmed that reducing only a single disulfide was sufficient to deduce the majority of the disulfide bonding pattern. Following alkylation to prevent the two free sulfhydryls from reforming a disulfide bond, proteolytic digestion and LC-MS/MS were used to identify peptide fragments containing disulfides **1–2** and **4–5** (Figure 1, Figure S4). Fragmentation data for the peptide containing Cys-6,7,8 did not support alkylation of Cys 8, and because it is extremely rare for adjacent residues to form a disulfide bond [49], there is no evidence to support a pattern containing **3–8** and **6–7** bonds. Consequently, the disulfide pattern of PMF-G was deduced to be either **1–2, 3–6, 4–5, 7–8** or **1–2, 3–7, 4–5, 6–8**, both differing from the canonical TFP pattern (**1–3, 2–4, 5–6, 7–8)**. Further LC-MS/MS analyses were unable to resolve the ambiguity between these two patterns, and additional structural characterization was limited by the availability of natural pheromone extract.

### Expression of rPMF-G

To fully characterize the structure of PMF-G fully, we sought to generate a correctly folded recombinant protein (rPMF-G). Heterologous expression of nearly all TFPs has relied on *in vitro* disulfide formation from scrambled products generated in *E. coli* or by solid phase synthesis [50], [51]. Based on the methods of Greenwald et al. [52], we employed the yeast system *Pichia pastoris*. Assembly PCR [35] was used to prepare a codon-optimized *pmf-g* gene for *P. pastoris*, which was successfully cloned into pPICZαA for targeted secretion into the growth media. *P. pastoris* clones were initially screened for successful transformation by colony PCR, and small-scale cultures were prepared for positive clones from both Mut+ (X33) and Mut^S^ (KM71H) backgrounds. rPMF-G was only secreted by clones in the Mut^S^ backround. All assays indicated that rPMF-G had a structure identical to the natural pheromone: LC/MS-MS and ESI-MS confirmed the sequence and mass, respectively; 5µg aliquots of PMF-G, rPMF-G, and a 1∶1 mixture of the two produced single peaks by RP-HPLC with retention times varying by <0.01 min; far UV circular dichroism (CD) spectroscopy generated nearly identical spectra for both proteins; and rPMF-G was validated to have the **1–2** and **4–5** disulfides (Figure S5). The literature suggests that this is only the second time a recombinant TFP has been synthesized without *in vitro* refolding [52].

### NMR Analysis of rPMF-G

**Figure 2:**
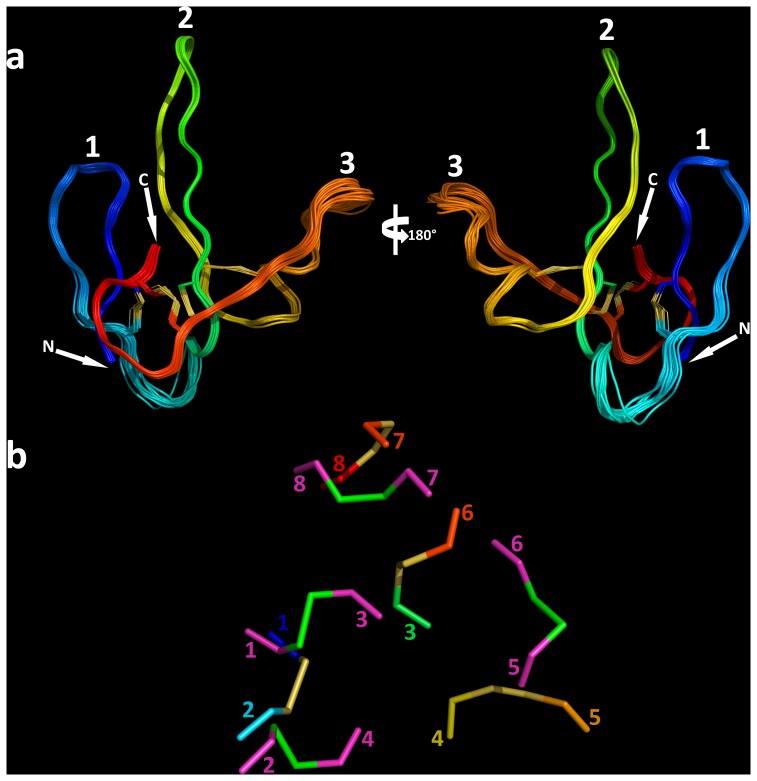
NMR-derived structural ensemble of PMF-G. (a) Backbone model of PMF-G with the twenty lowest-energy conformers, color coded from N- to C-terminus (blue to red), and peptide finger numbers denoted (1–3); (b) disulfide bonds in PMF-G from underside view (same color scheme as a) and a representative TFP (1IQ9, carbons in magenta, sulfurs in green).

**Figure 3:**
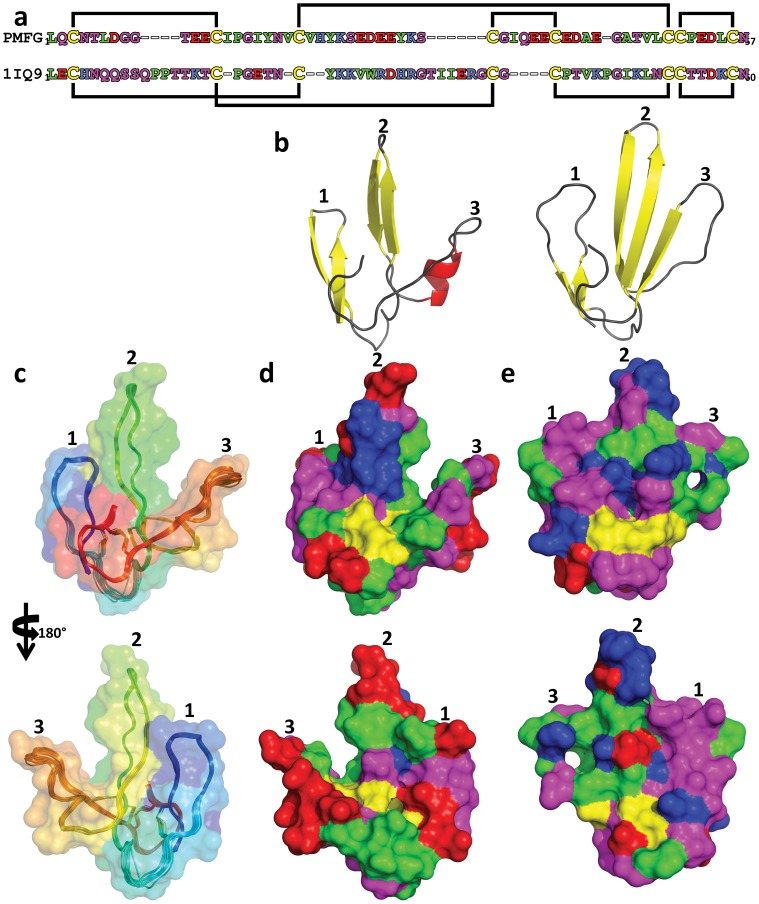
Surface models of PMF-G. (a) Alignment of PMF-G with a representative TFP (1IQ9), color coded by residue type (acidic, red; basic, blue; hydrophilic, purple; nonpolar, green; cysteine, yellow), with disulfide bonds denoted by the black lines; (b) secondary structure schematic comparing PMF-G (left) and a representative TFP (right; 1IQ9); (c) backbone model of PMF-G (20 lowest-energy conformers) with partially transparent surface rendering (both color coded N- to C-terminus, blue to red); (d) full surface rendering of PMF-G color coded by residue type (same color code as a); (e) surface rendering of 1IQ9 (same color code as a).

**Figure 4:**
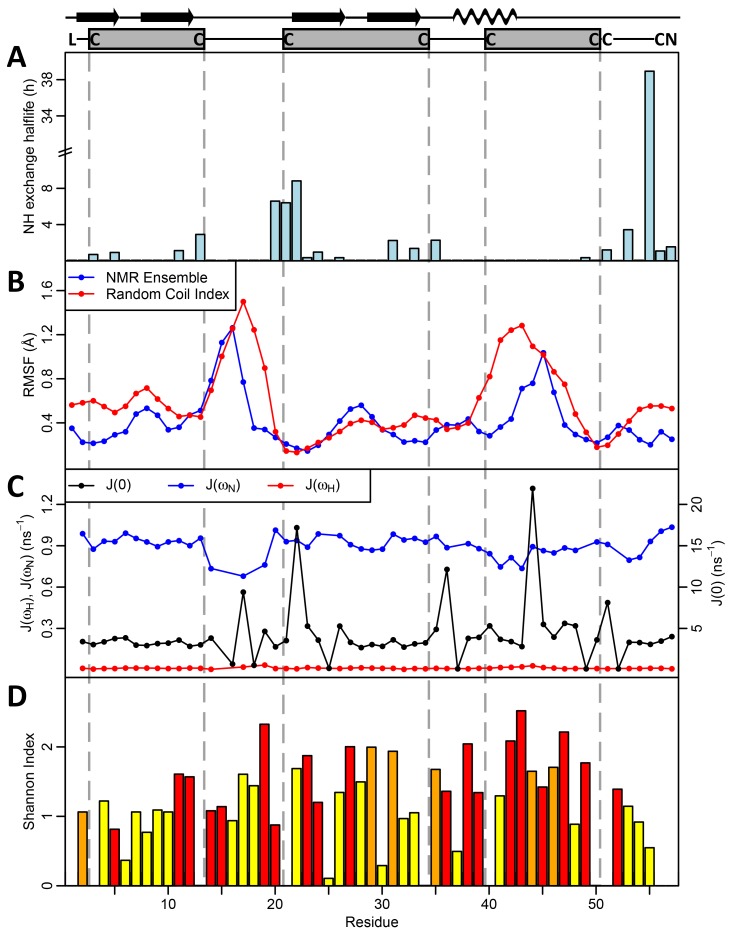
Measurements of structural and sequence variability in PMF. (a) Backbone amide (H_N_) exchange H/D exchange rates measured by half life (in hours), with proline residues omitted; (b) Root mean squared fluctation (RMSF) per residue in the PMF structural ensemble (blue) and predicted from the random coil index (red); (c) spectral density functions at 0, ω_N_, and ω_H_; J(0) is sensitive to fast (ns) and slow (µs-ms) motions, J(ω_N_) to motiions on time scales faster than (1/ω_N_ = 2 ns), and J(ω_H_) to motions faster than ^1^H (1/ω_H_ = 0.2 ns); (d) Sequence variability (Shannon entropy index) at each residue measured for all Class I PMFs, shaded according to likelihood of positive selection at each position (red p<0.01, orange p<0.05; yellow = neutral selection). Seven out of the nine non-conserved amino acids in finger 3 display signatures of positive selection, suggesting combined structural flexibility and rapid evolution in this region.

To determine the solution structure of PMF-G, milligram quantities of ^15^N-labeled rPMF-G were prepared for multidimensional NMR analysis. Essentially complete assignments of all backbone atoms and side-chain atoms were obtained from 2D NOESY, TOCSY, 3D ^15^N HSQC-NOESY, HNCO and natural abundance ^1^H(^13^C) HSQC experiments. The 3D structure of PMF-G was solved using standard restrained molecular dynamics simulation with distance restraints determined by nuclear Overhauser effect (NOE) measurements and dihedral angle restraints using TALOS+ (Table 1). Additional hydrogen bond constraints were determined by measuring hydrogen/deuterium exchange rates for backbone amide protons (Figure S6). Structure calculations were performed with constraints using the two alternative disulfide patterns; multiple van der Waals and distance violations were observed when the **3–7/6–8** disulfides were included, whereas there were no consistent conflicts in the other model. Thus, we concluded that the disulfide-bonding pattern of PMF-G is **1–2, 3–6, 4–5, 7–8**. Surprisingly, despite shuffling in 3 of the 4 disulfides, PMF-G still adopts an overall “three-finger” shape (Figure 2). However, the resulting adjustments in the protein backbone eliminated much of the classical TFP topology (a two-stranded β-sheet in finger 1 and a three-stranded β-sheet in finger 2 and finger 3) (Figure 3B). This includes the loss of a finger 3 β-strand, leading to a two-stranded sheet in finger 2, and a rotation in finger 2 such that it is near-orthogonally aligned with finger 1 (likely a result of the self-contained altered **3–6** and **4–5** disulfides). Additionally, the novel disulfide pattern eliminated the conserved van der Waals network present between the canonical **1–3, 2–4, 7–8** disulfides, which stabilize the base of the classical TFP structure [32] (Figure 2B). Analysis of side chain properties revealed a general segregation between the two elongated protein faces: one side contained the majority of negative and hydrophobic residues, while the other contained the few positive charges and additional hydrophilic residues. This highly polarized negative charge density is in sharp contrast to most other TFP members that typically have a net positive charge (Figure 3D–E). The lack of secondary structure between fingers 2 and 3 may be the result of charge repulsion due to the concentration of acidic residues on the two fingers, leading to an extended finger 3 and forming a cleft between them. The majority of backbone amides in PMF-G were solvent accessible and rapidly exchanged (34 out of 54 H_N_ groups were undetectable after 20 minutes in D_2_O, and only 13 H_N_ groups had half-lives greater than one hour; Figure S6); however, the slowest-exchanging amides were found near the base of finger 2, and near the N- and C-termini nexus. Notably, the Leu55 amide proton was nearly non-exchangeable (half-life >38 hours) (Figure 4A), and is likely H-bonded with the Gln2 backbone carbonyl. Additionally, two highly conserved residues on finger 2 (Glu30 and Lys25) were found in close proximity, and likely form a novel salt bridge that help stabilize finger 2. Although the resulting structural model is well determined (average backbone rmsd = 0.31 Å), there is less β-sheet structure in PMF-G relative to other TFPs (Figure 3B; consistent with CD results, Figure S5B). When compared against all available PDB sequences using PDBeFold, the closest match was a γ-bungarotoxin (1MR6) with a Q-score of only 0.24 (P-score 0.1, Z-score 1.2), suggesting that PMF has a previously uncharacterized protein topology.

**Table 1:** Restraints and statistics of the PMF-G structural ensemble.

Structural constraints				
*NOE distances*:	<3.00 Å	78 (11.1%)	Intraresidue	161 (22.9%)
	3.00–3.99 Å	239 (33.9%)	Adjacent (|i−j| = 1)	217 (30.8%)
	4.00–4.99 Å	245 (34.8%)	Short (1<|i−j|≤5)	110 (15.6%)
	5.00–5.50 Å	142 (20.2%)	Long (|i−j|>5)	216 (30.7%)
	Total	704	Total	704
*Dihedral angles*:	76			
*Hydrogen bonds*:	3			
*Disulfide bonds*:	4			
**Structural statistics**				
*Average RMSD to mean (Å)*			*Ensemble (n = 20)*	*Lowest energy*
	Backbone		0.31±0.08	0.19
	Heavy atom		0.73±0.07	0.64
*Target function*			0.49±0.052	0.37
*Violations*	Upper limit	#	1±1	0
		rms	0.0060±0.0015	0.0039
		max	0.13±0.06	0.07
	van der Waals	#	2±0	2
		sum	2.3±0.2	1.9
		max	0.23±0.03	0.20
	torsion angles	#	0±0	0
		rms	0.7072±0.0562	0.6587
		max	2.66±0.30	2.54
*Ramachandran statistics (Procheck * [*72*] *)*				
	Most favored region (%)		88.9±0.02	87.5%
	Additionally allowed regions (%)		9.1±0.02	10.4%
	Generously allowed regions (%)		2.1±0	2.1%
	Disallowed regions (%)		0±0	0

### Comparative Modeling with Additional PMF Isoforms

**Figure 5:**
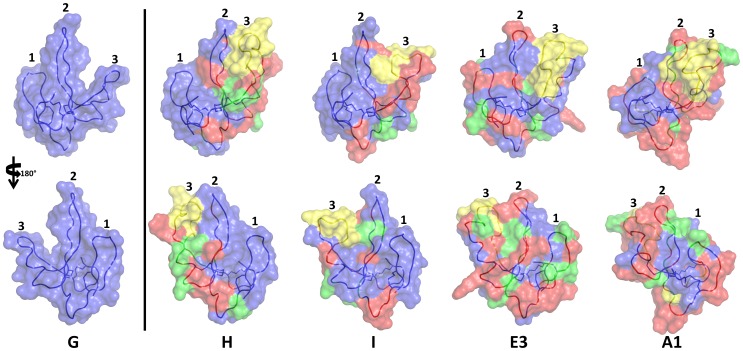
Homology modeling of major PMF isoforms. Homology models of four additional PMF isoforms that are highly expressed in *P. shermani* (isoform H, accession #JF274289; isoform I, accession #JF274304; isoform E3, accession #JF274344; isoform A1, accession #JF274380). Models are color coded according to amino acid conservation relative to PMF-G, which is included as a reference in the first panel (same residue, blue; conservative substitution, green; nonconservative substitution, red; insertion, yellow).

**Figure 6:**
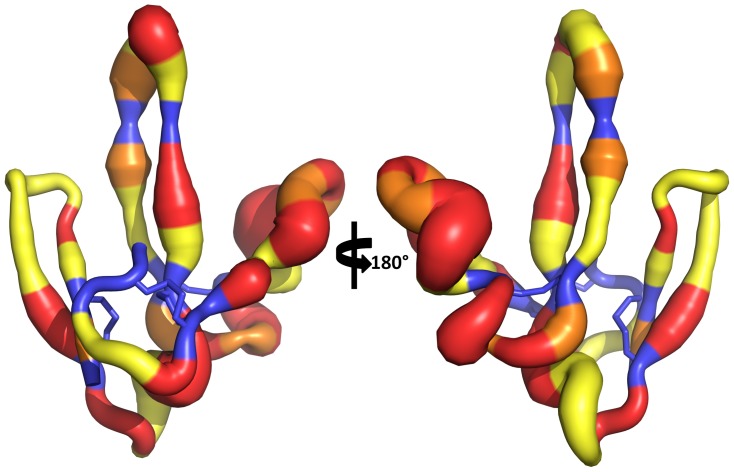
Rates of molecular evolution on PMF-G. Putty model of PMF-G, with backbone width proportional to residue variability (Shannon-Weaver index in Figure 4D), and color-coded according to the likely mode of molecular evolution (based on data from Wilburn et al. [20]; backbone, black; purifying selection, blue; neutral selection, yellow; positive selection, 0.01≤p<0.05, orange; positive selection, p<0.01, red).

In order to assess how PMF sequence hypervariability may be structurally manifested, sequence comparison and homology modeling were conducted for additional PMF isoforms using the NMR-derived PMF-G structure as a template. Of the 99 PMF haplotypes reported in Wilburn et al. [20], the spacing of the first 5 cysteines is conserved in 75% of the sequences, and varies by no more than 3 residues in the remaining 25%. This could be considered an underestimate, as 86% of Class I PMFs (which comprise ∼90% of the total PMF protein) share this spacing for the first 5 cysteines. However, the region between the 5^th^ and 6^th^ cysteines (equivalent to most of finger 3) is more variable, both in length (15.6±2.6 residues; PMF-G = 9 residues) and sequence. Homology models for four additional highly expressed PMF isoforms (H, I, E3, and A1) all have extended loops on finger 3 (Figure 5). In the three most abundant Class I PMFs (G, H, and I), fingers 1 and 2 are predicted to be nearly identical with respect to both sequence and structure, with finger 3 being the only highly variable region. Additionally, in the PMF-G structural ensemble, two regions display greater backbone flexibility than the rest of the protein: the loop between fingers 1 and 2, and the length of finger 3. This flexibility is further supported by multiple lines of evidence: nearly all of the backbone amides in these regions are solvent accessible and exchanged rapidly, fewer well-defined NOEs were observed for these regions, ^15^N linewidths and measured R_2_ values were larger for many of the backbone amides, and these residues had higher predicted random coil indexes (based on chemical shift values) (Figure 4A–B; Figure S7). Relaxation experiments confirmed that residues in these regions (specifically, 17, 36, 44, 45, 47, and 51) showed flexibility on µs-ms time scales (Figures 4C; Figure S8). Additionally, models of molecular evolution (from Wilburn et al. [20]) indicated that the majority of positively selected residues are located in on finger 3 (Figures 4D and 6). Together, these data suggested that, in addition to rapid evolution of finger 3, the altered disulfide bonding pattern of PMF-G disrupted the classical TFP topology and permitted greater structural flexibility in this finger in order to maximize the number of sequence/structural permutations of PMF.

## Discussion

Within evolutionary, biomedical, and structural scopes, the TFP superfamily has remained a key research target due to both the unique structural elements and the diverse functions of its many orthologs. Within the snake toxin TFP family alone, through adaptive evolution, members have adopted many distinct functions including ion channel blocking [53]–[55], nicotinic and muscarinic receptor antagonists [23], [56], [57], acetylcholinesterase inhibition [58], cell-adhesion regulation [59], integrin binding [60], and pore formation in the plasma membrane [61], [62]. There also exist membrane-bound TFPs, including CD59, Ly6 antigen, and the newt regenerative positional maker Prod1. In a study by Garza-Garcia et al. [27], the solution structure of Prod1 was solved and fit within the canonical TFP framework; however, within a phylogenetic context, Prod1 was much more similar to PMF in both sequence and predicted biochemical characteristics. If PMF and Prod1 are relatively recent paralogs within salamanders, then PMF’s novel topology and disulfide pattern are likely more recently derived characteristics. Notably, expression of PMF-G in *P. pastoris* suggested that the novel disulfide bonding pattern is thermodynamically favorable and not the product of plethodontid-specific chaperones and/or protein disulfide isomerases. In contrast to most TFPs (excluding Prod1), PMF has a high net negative charge which may affect its folding dynamics. A comprehensive structural analysis of the TFP superfamily by Galat et al. [32] found little sequence similarity beyond the 8 core cysteine residues, and last three Cys arranged in the CCXXXXCN motif. Despite the lack of sequence similarity, three of the four disulfides (**1–3**, **2–4**, **7–8**) form a tight van der Waals interaction network that stabilize the double β-sheet structure (<4 Å between the bonds, <1 Å average rmsd for this network between TFP members). In the example where the Cys 6–7 doublet is split by an additional residue (TGFβ-RII), this network is disrupted and the structure is less stable. Likewise, the altered disulfide bonding pattern of PMF-G disrupted this network such that the disulfides are spaced further apart (Figure 2B), and may partially relate to the loss of the β-strand normally found in finger 3. Interestingly, the spacing between the first two cysteines was conserved among all PMFs (9 residues), and is shorter than any structurally characterized TFP (length = 17.0±5.9 residues; min residues = 10). This shorter spacing may be important in promoting formation of the **1–2** disulfide, which in turn could prevent the canonical **1–3** bond from forming and help drive the novel disulfide pattern.

A central question that remained was what is the adaptive value in PMF adopting a novel disulfide-bonding pattern relative to the canonical TFP structure? Within ∼30 million years, the PMF complex has undergone tens to hundreds of gene duplications to yield the ∼100 expressed mRNA sequences observed in *P. shermani* cDNA [20]. At the same time, these genes have been under strong sexual selective pressure to differentiate and adopt potentially novel signaling roles in order to affect female behavior and physiology [20], [22]. When whole mental gland extract was applied to female salamanders, courtship time decreased by ∼20% [16]. Surprisingly, when a mixture of more than 30 PMF isoforms was tested (that did not include PMF-G), courtship time increased [63]. While this subset of PMF isoforms activated VNO neurons and regions of the female brain classically involved in pheromone response [17]–[19], more recent set of experiments revealed that a more complete PMF mixture (that included PMF-G) decreased courtship time similar to whole extract but without significantly activating more VNO neurons (Wilburn, Houck, Woodley, and Feldhoff, unpublished data). Consequently, our working hypothesis is that synergistic interactions between the many diverse isoforms are necessary for PMF to increase female mating receptivity. This is perhaps in contrast to other polygenic pheromone families, such as MUPs in mice, where different isoforms are uniquely involved in mediating gender recognition, male-male aggressive behavior, female sexual receptivity, and learning of individual odor profiles [9], [10], [64]–[66]. Expression of different PMF isoforms is highly variable between male salamanders; however, PMF always constitutes ∼50% of the total mental gland pheromone [21], with PMF-G almost always being the most abundant isoform (∼12% of the total PMF) [20]. In the current study, we have provided evidence that the most variable and rapidly evolving segment of PMF (finger 3) is also structurally flexible. Homology modeling supported that both the length and shape of this finger is likely variable in additional PMF isoforms, and that this segment has the greatest topological differences from the canonical TFP structure. Combining the sequence variability, structural flexibility, and altered topology relative to the TFP superfamily, we hypothesize that finger 3 plays a critical role in PMF-receptor interactions, utilizing both residue variability and backbone flexibility to permit a significantly greater number of structural permutations that may occupy a broader range of female receptors. As female receptors continue to evolve, this structural flexibility may still permit PMF to interact with target receptors by adopting a slightly different conformation, without the need for complementary mutations. Consequently, we hypothesize that PMF may have evolved a form of “resilience” to mutations in female receptors, that might otherwise ablate pheromone:receptor interactions, and thus provide males with an enhanced ability to stimulate any mating female in the breeding population.The precise mechanism by which PMF regulates female mating behavior has yet to be determined; however, PMF stimulated neurons in the female vomeronasal organ and activated regions of the brain known to be involved in pheromone response [18], [19]. Based on these data, PMF is presumably binding to a vomeronasal type-2 receptor (V2R), which are highly abundant in the *P. shermani* VNO [67] and have been implicated in protein pheromone signaling in rodents [9], [68], [69]. Very few specific receptor:ligand pairs have been identified for vomeronasal receptors [11], [69]–[71], but to date, none of these examples include TFP:V2R interactions. Recently, the 3D structure was determined for the mouse sex pheromone ESP1, and through mutagenesis assays and molecular docking studies with its specific V2R receptor, it was determined that charge-charge interactions provide most of the binding specificity. Future studies of PMF will seek to determine specific VNO receptors that mediate reception of plethodontid courtship pheromones and understand the molecular interactions that drive pheromone:receptor co-evolution.

## Conclusions

Over tens of millions of years, sexual selection has promoted rapid evolution in the three-finger protein pheromone, Plethodontid Modulating Factor. In addition to sequence hypervariability, this process has altered the highly conserved TFP disulfide bonding pattern and topology in order to increase backbone flexibility in the putative receptor binding sites. Taken together, the sequence diversity and structural flexibility likely permit thousands of PMF conformers, increasing both the signal plasticity of PMF and the likelihood of stimulating any female in the mating population. In support of this hypothesis, preliminary data suggest that female receptivity increases when females receive sufficient PMF isoform diversity. This “evolved conformational flexibility” may confer PMF robustness to ever evolving changes in female receptors. This work lays the foundation for future research in understanding the molecular adaptations that arise as part of the sexual conflict between males and females that can lead to an evolutionary “arms race” of signals by one gender and receptors of the other gender.

## Supporting Information

Figure S1 **Purification scheme of natural PMF-G.** (A) Initial separation of whole *P. shermani* pheromone extract by strong anion exchange HPLC with the mixed rate gradient. Fractions E-I described in Wilburn et al. (2012) corresponding to PMF were pooled (elution fractions 43–57 min). (B) Following sample concentration, the PMF mixture was further purified using size-exclusion chromatography. (C) The size exclusion chromatography samples were re-separated by strong anion exchange HPLC on a shallow linear gradient with fraction G collected (∼42 min). (D) Fraction G was subjected to a second round of strong anion exchange HPLC, and (E) finally purified at >99% purity by RP-HPLC. (F) MS analysis of PMF-G revealed a highly enriched signal at the expected average mass of 6256 Da.
(JPG)

Figure S2 **PMF-G contains 4 disulfide bonds.** (A) Treatment of PMF-G with IAA resulted in no CAM alkylation, unless first reduced with DTT, implying that all cysteine residues are disulfide bonded in the intact protein; (B) Similar treatment of rPMF-G confirmed that both its molecular weight and cystine content are identical to natural PMF-G.
(TIF)

Figure S3 **Partial reduction of PMF-G.** RP-HPLC separation of PMF-G treated with TCEP at low pH to induce restricted disulfide reduction. Each peak is labeled with the number of remaining disulfides, with increasing hydrophobicity as the number of free sulfhydryls increases.
(JPG)

Figure S4 **Mass spectral analysis of partially reduced PMF-G.** Sample ion spectra of PMF-G, partially reduced with TCEP, the 3 disulfide bonded species collected by RP-HPLC, free sulfhydryls alkylated by iodoacetamide to add a CAM group, and proteolytically digested using chymotrypsin. Specific masses of PMF that were essential for disulfide bond deduction are labeled.
(TIF)

Figure S5 **Comparison of native and recombinant PMF-G.** (A) RP-HPLC analysis comparing 5 µg aliquots of PMF-G, rPMF-G, and equal amounts of PMF-G and rPMF-G. The similarity in retention times strongly suggested identical structures between native and recombinant PMF-G. (B) Far UV CD analysis of native and recombinant PMF-G produced very similar spectra, with estimated secondary structure of ∼11% α-helix and ∼29% β-sheet content (K2D3; 27), which is similar to NMR results measured by DSS in Pymol (11% α-helix, 33% β-sheet) [73], [74]. (C) Both PMF-G and rPMF-G were treated with TCEP for 20min and major peaks represent 4, 3, 2, 1, and 0 intact disulfides. Retention times were slightly adjusted to correct for run-to-run variation (∼0.6 min, 2 different RP-C18 columns, ∼2 weeks apart). Data from mass spectral analysis of the 1 reduced disulfide species are consistent with the results in Figure 1.
(TIF)

Figure S6 **PMF-G amide H-D exchange rate.** Plot of peak integration versus time of ^15^N-HSQC spectra recorded every hour over 24 hrs for rPMF-G lyophilized and dissolved in D_2_O. An exponential decay curve (v = v_0_exp(-kt)) was fitted to all peaks with 3 or more points.
(TIF)

Figure S7 **PMF-G backbone amide ^15^N linewidths.** Barplot of ^15^N linewidths for backbone amides derived from a ^15^N-HSQC spectrum. The N-terminal Leu and two Pro residues were assigned 0 Hz, and residues undectable by ^15^N-HSQC (residues 16, 18, 25, 37, 49) were assigned 27.5 Hz.
(TIF)

Figure S8 **NMR relaxation analysis.** Relaxation analysis of rPMF-G examined by (a) ^15^N[^1^H] steady-state heteronuclear NOE measurements (with lower values suggestive of conformational changes), (b) spin-lattice (longitudinal) relaxation rate constants (R_1_) (with lower values indicating sub-ns exchanges), (c) spin-spin (transverse) relaxation rate constants (R_2_) (with higher values indicating µs-ms exchanges), and (d) the R_ex_ rate. R_1_ and R_2_ are reported as parameter estimates ±95% confidence interval.
(TIF)
