# Supplementary figures and images for: Structural Insights into the Evolution of a Sexy Protein: Novel Topology and Restricted Backbone Flexibility in a Hypervariable Pheromone from the Red-Legged Salamander, Plethodon shermani
Source: PLoS One. 2014 May 21;9(5):e96975. doi: 10.1371/journal.pone.0096975 (PMC4029566; doi:10.1371/journal.pone.0096975)

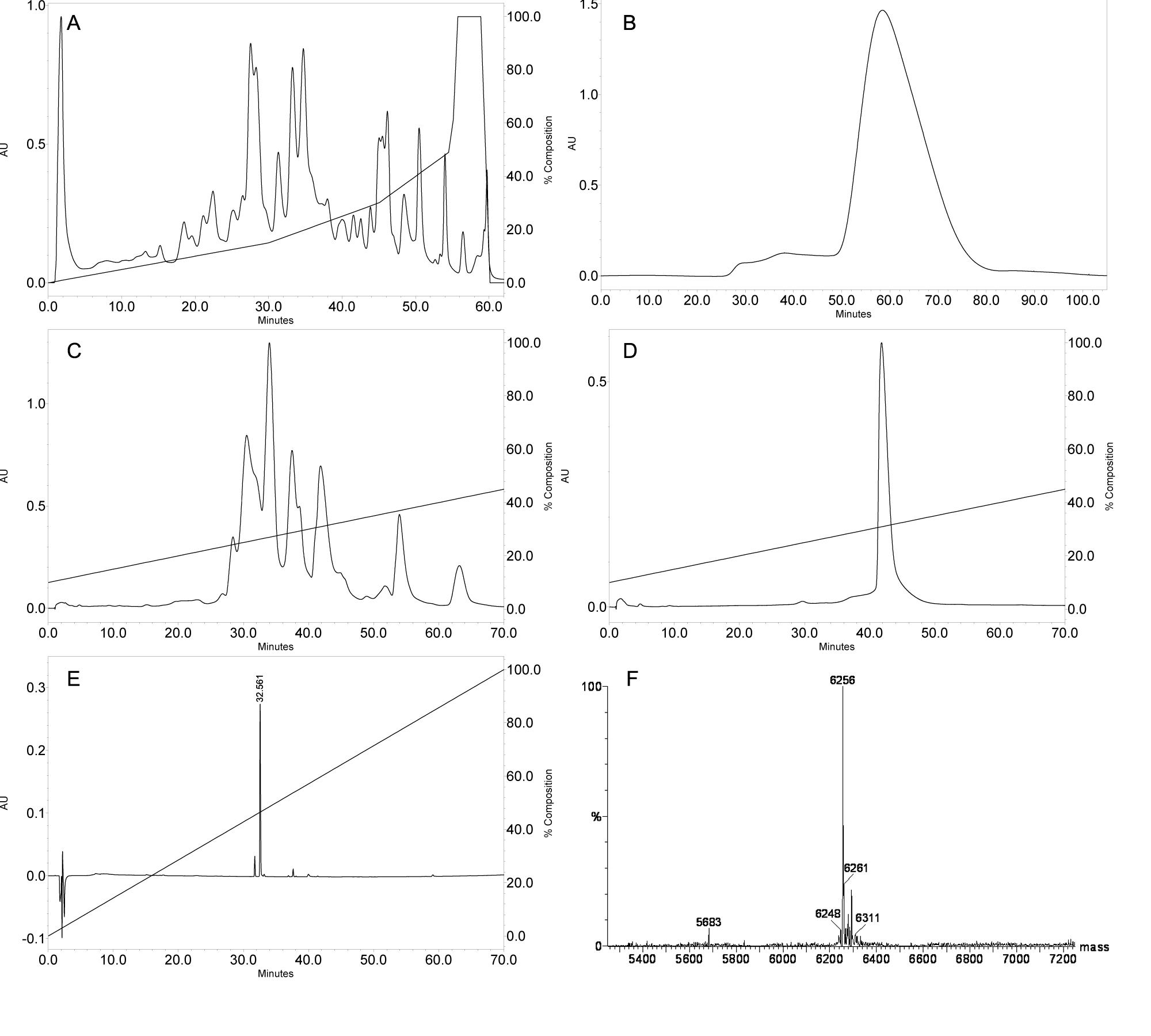

Supplement: Figure S1 — Purification scheme of natural PMF-G. (A) Initial separation of whole P. shermani pheromone extract by strong anion exchange HPLC with the mixed rate gradient. Fractions E-I described in Wilburn et al. (2012) corresponding to PMF were pooled (elution fractions 43–57 min). (B) Following sample concentration, the PMF mixture was further purified using size-exclusion chromatography. (C) The size exclusion chromatography samples were re-separated by strong anion exchange HPLC on a shallow linear gradient with fraction G collected (∼42 min). (D) Fraction G was subjected to a second round of strong anion exchange HPLC, and (E) finally purified at >99% purity by RP-HPLC. (F) MS analysis of PMF-G revealed a highly enriched signal at the expected average mass of 6256 Da. (JPG) [file pone.0096975.s001.jpg]

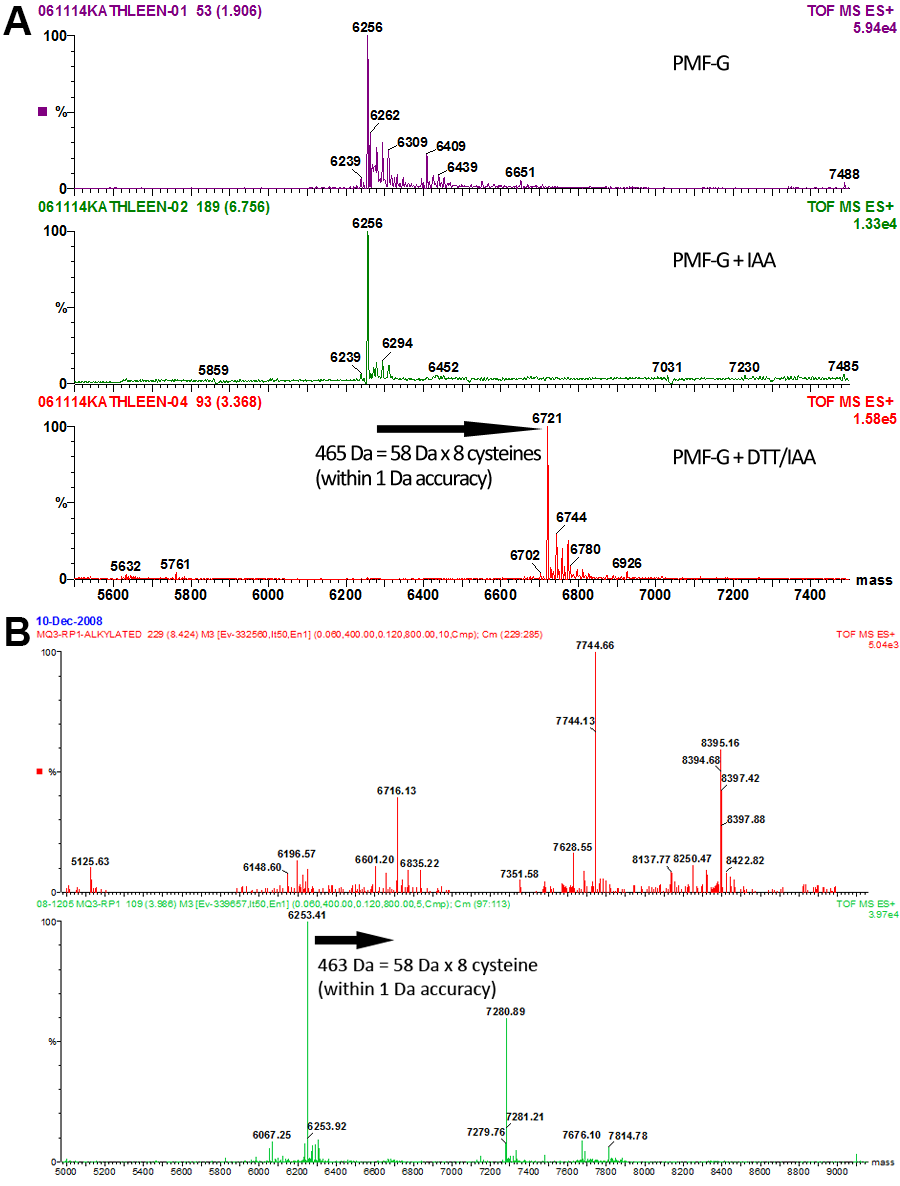

Supplement: Figure S2 — PMF-G contains 4 disulfide bonds. (A) Treatment of PMF-G with IAA resulted in no CAM alkylation, unless first reduced with DTT, implying that all cysteine residues are disulfide bonded in the intact protein; (B) Similar treatment of rPMF-G confirmed that both its molecular weight and cystine content are identical to natural PMF-G. (TIF) [file pone.0096975.s002.tif]

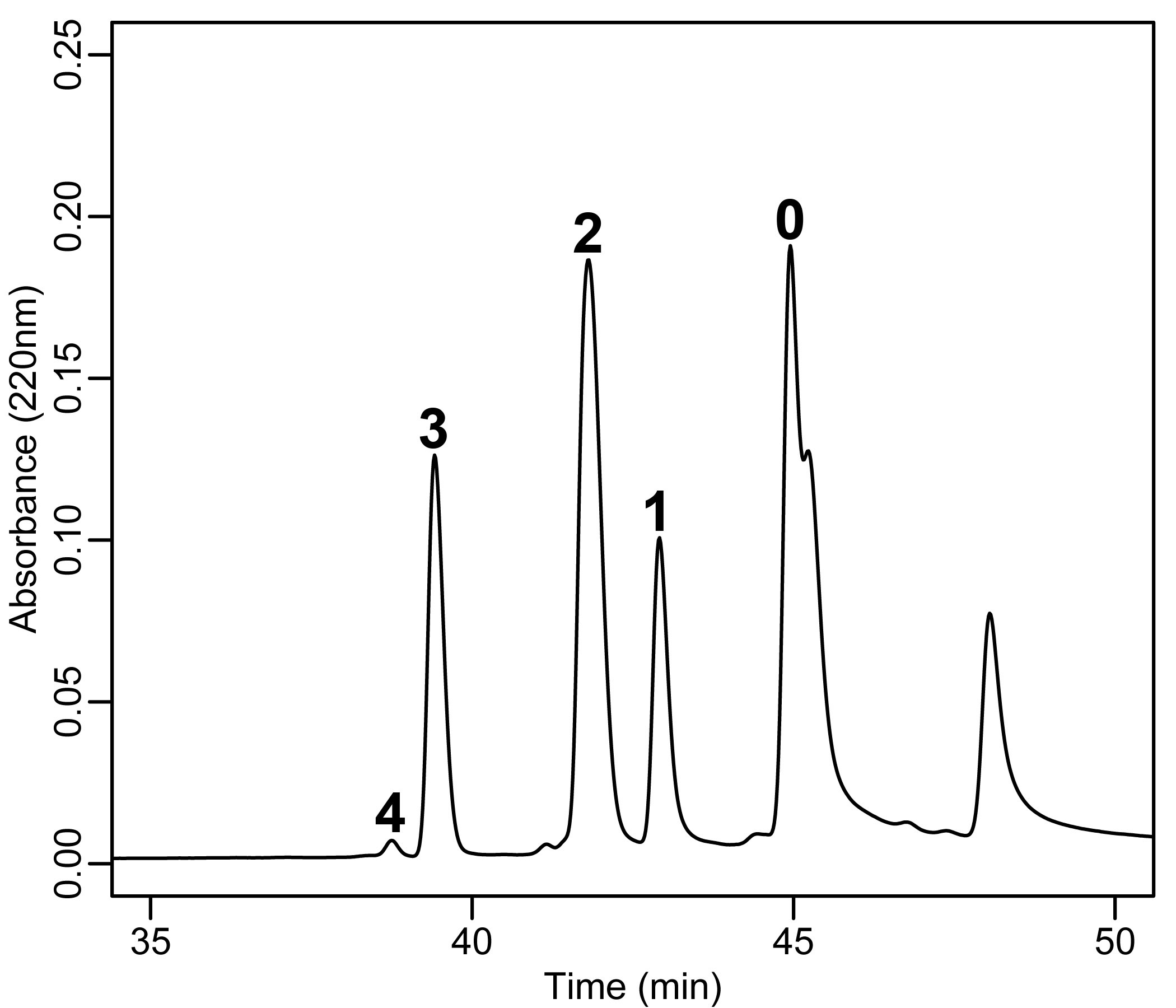

Supplement: Figure S3 — Partial reduction of PMF-G. RP-HPLC separation of PMF-G treated with TCEP at low pH to induce restricted disulfide reduction. Each peak is labeled with the number of remaining disulfides, with increasing hydrophobicity as the number of free sulfhydryls increases. (JPG) [file pone.0096975.s003.jpg]

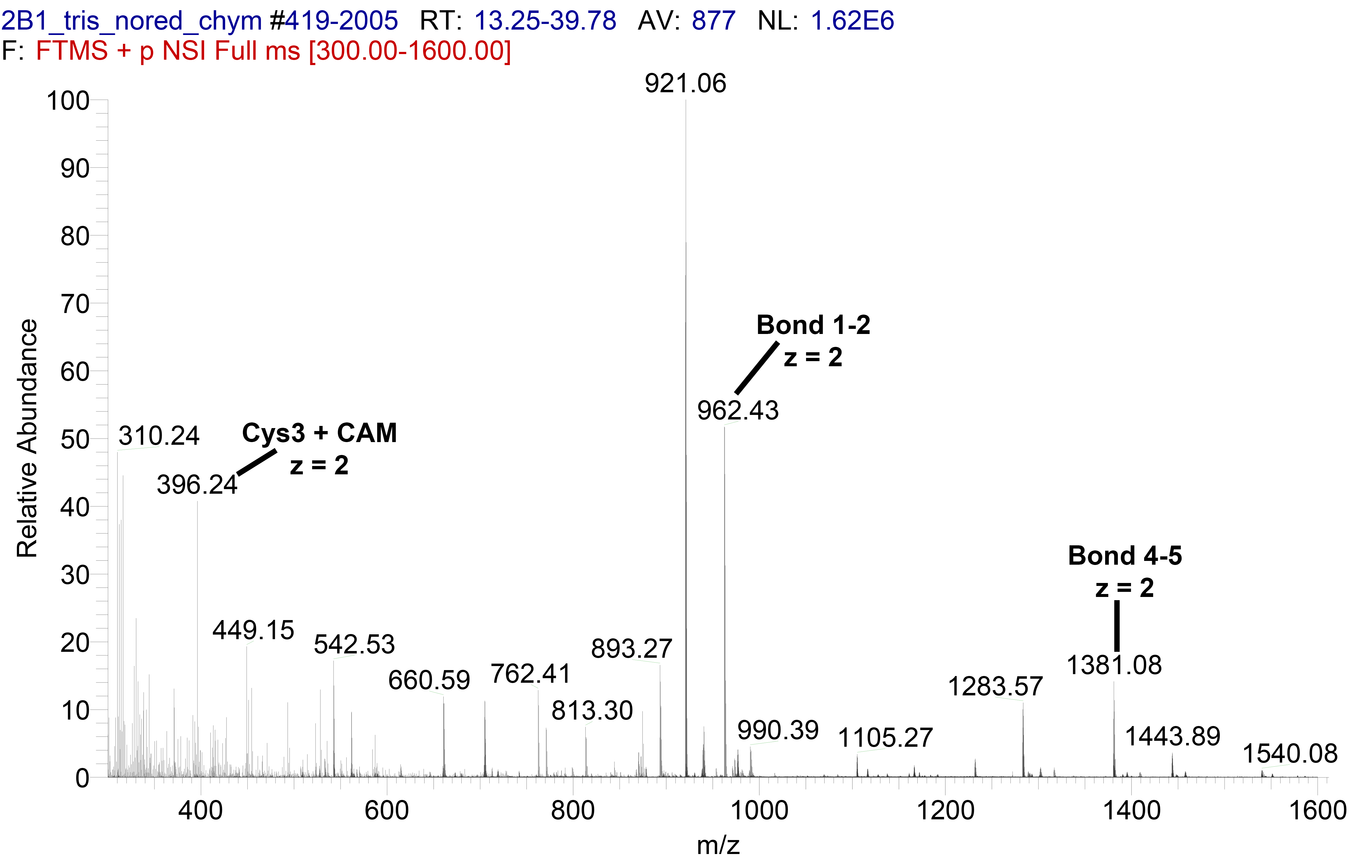

Supplement: Figure S4 — Mass spectral analysis of partially reduced PMF-G. Sample ion spectra of PMF-G, partially reduced with TCEP, the 3 disulfide bonded species collected by RP-HPLC, free sulfhydryls alkylated by iodoacetamide to add a CAM group, and proteolytically digested using chymotrypsin. Specific masses of PMF that were essential for disulfide bond deduction are labeled. (TIF) [file pone.0096975.s004.tif]

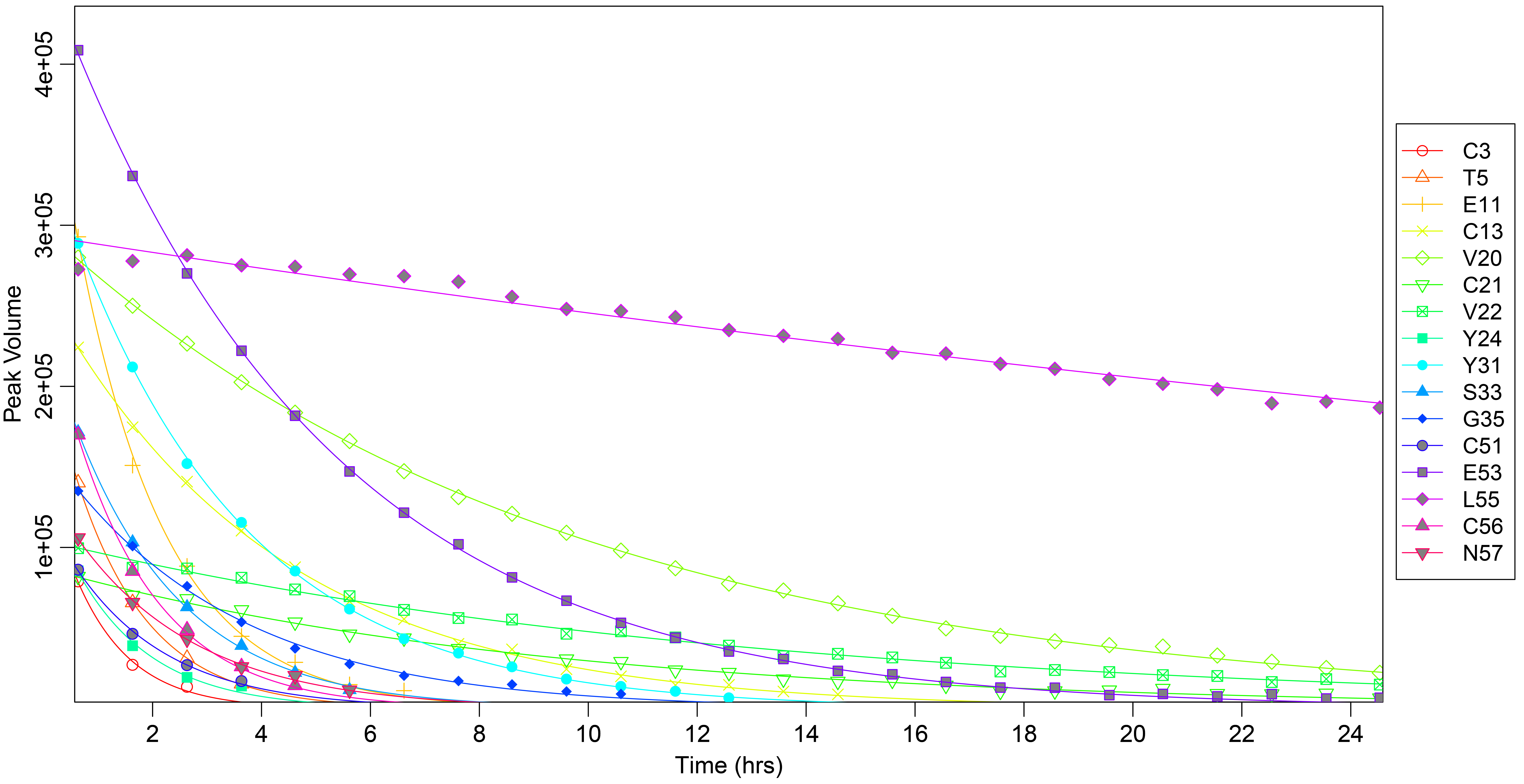

Supplement: Figure S6 — PMF-G amide H-D exchange rate. Plot of peak integration versus time of 15N-HSQC spectra recorded every hour over 24 hrs for rPMF-G lyophilized and dissolved in D2O. An exponential decay curve (v = v0exp(-kt)) was fitted to all peaks with 3 or more points. (TIF) [file pone.0096975.s006.tif]

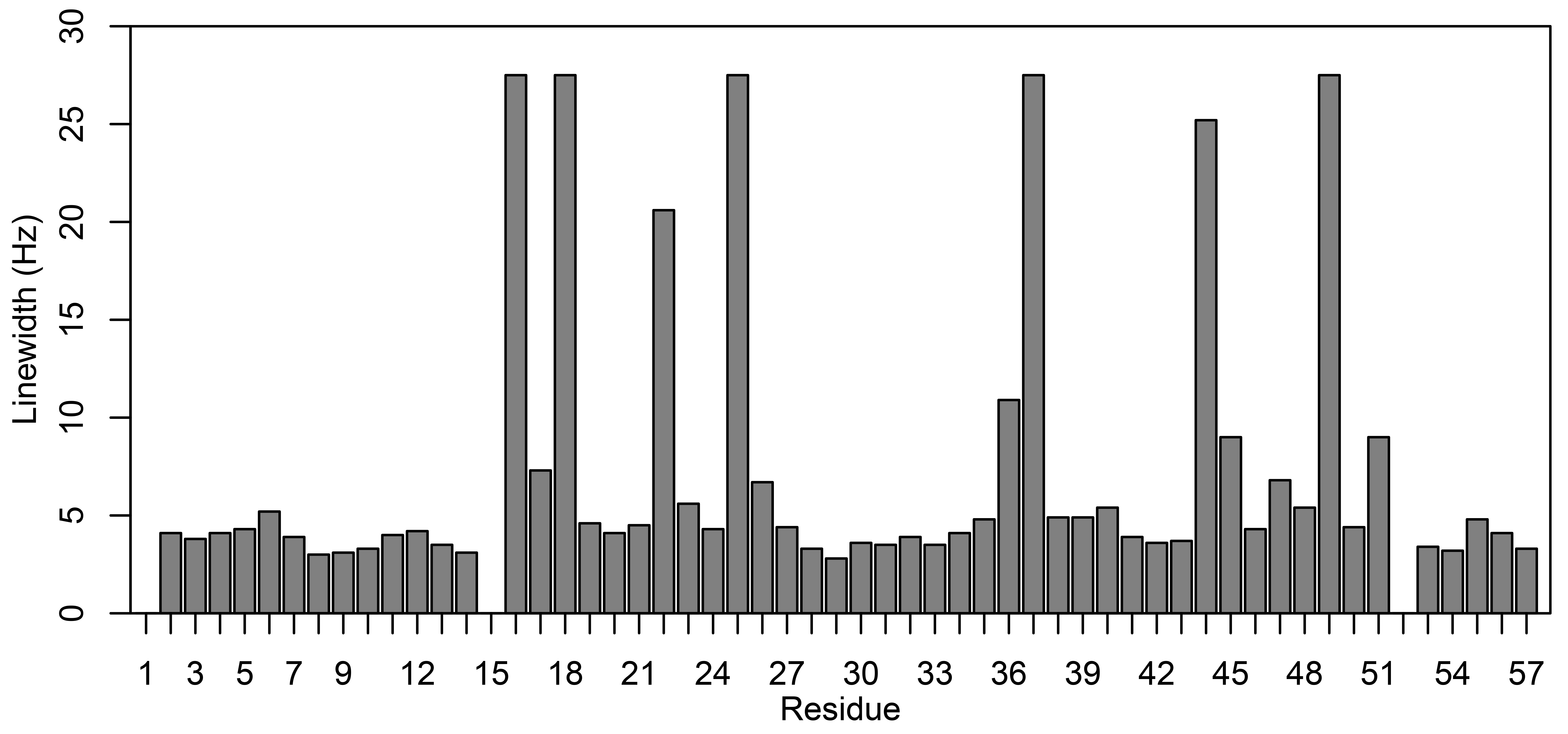

Supplement: Figure S7 — PMF-G backbone amide 15N linewidths. Barplot of 15N linewidths for backbone amides derived from a 15N-HSQC spectrum. The N-terminal Leu and two Pro residues were assigned 0 Hz, and residues undectable by 15N-HSQC (residues 16, 18, 25, 37, 49) were assigned 27.5 Hz. (TIF) [file pone.0096975.s007.tif]

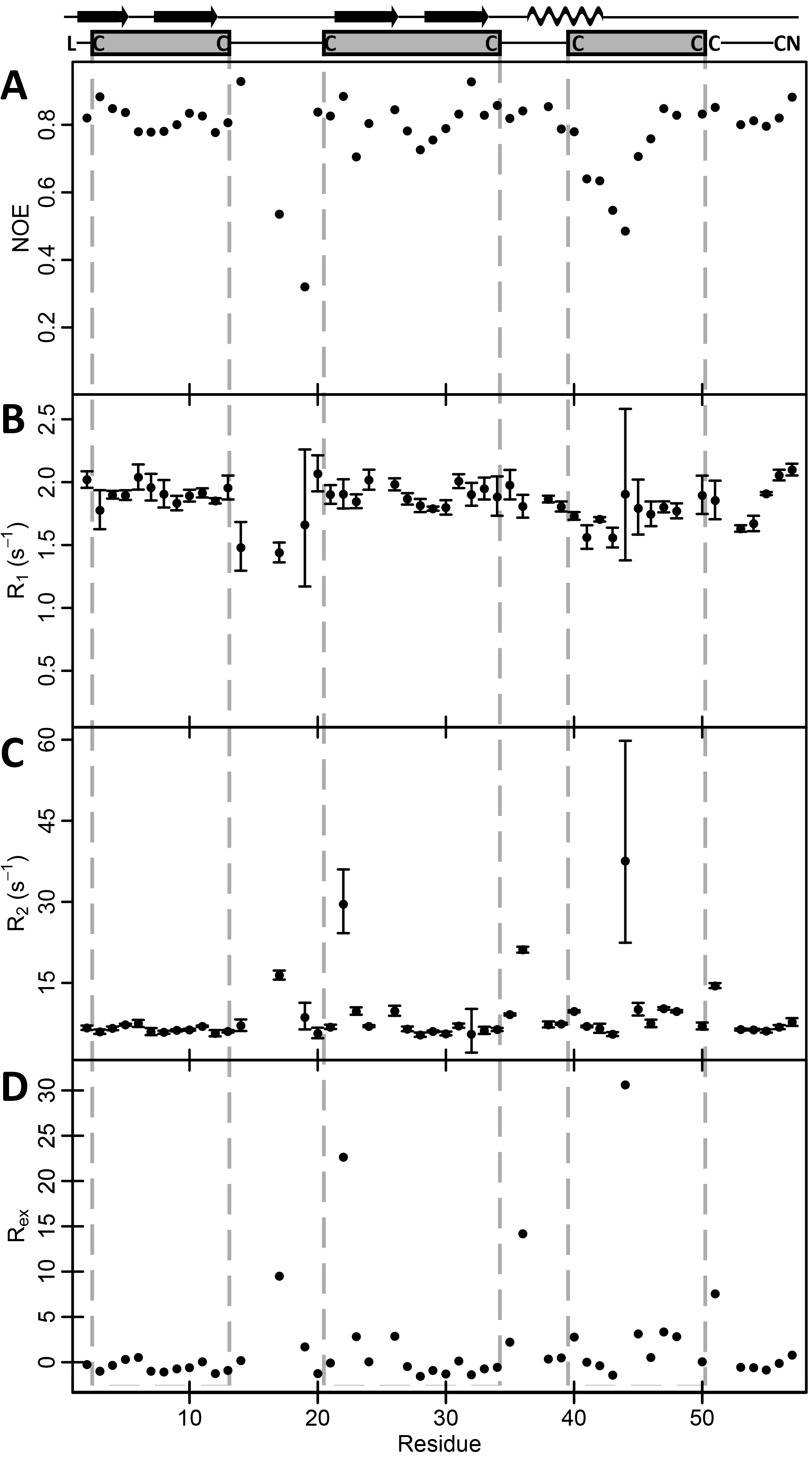

Supplement: Figure S8 — NMR relaxation analysis. Relaxation analysis of rPMF-G examined by (a) 15N[1H] steady-state heteronuclear NOE measurements (with lower values suggestive of conformational changes), (b) spin-lattice (longitudinal) relaxation rate constants (R1) (with lower values indicating sub-ns exchanges), (c) spin-spin (transverse) relaxation rate constants (R2) (with higher values indicating µs-ms exchanges), and (d) the Rex rate. R1 and R2 are reported as parameter estimates ±95% confidence interval. (TIF) [file pone.0096975.s008.tif]
